# Molecular properties of human guanylate cyclase–activating protein 2 (GCAP2) and its retinal dystrophy–associated variant G157R

**DOI:** 10.1016/j.jbc.2021.100619

**Published:** 2021-04-01

**Authors:** Anna Avesani, Valerio Marino, Serena Zanzoni, Karl-Wilhelm Koch, Daniele Dell'Orco

**Affiliations:** 1 Department of Neurosciences, Biomedicine and Movement Sciences, Biological Chemistry Section, University of Verona, Verona, Italy; 2 Centro Piattaforme Tecnologiche, University of Verona, Verona, Italy; 3 Department of Neuroscience, Division of Biochemistry, University of Oldenburg, Oldenburg, Germany

**Keywords:** guanylate cyclase (guanylyl cyclase), cGMP, calcium-binding proteins, phototransduction, retinal degeneration, vision, retina, neurodegenerative disease, GUCA1B, GCAP, ANS, anilinonaphthalene-1-sulfonic acid, CNG, cyclic nucleotide–gated channels, DLS, dynamic light scattering, GC, guanylate cyclase, GCAP, guanylate cyclase–activating protein, GCAP1, guanylate cyclase–activating protein 1, GCAP2, guanylate cyclase–activating protein 2, IRD, inherited retinal degeneration, ITC, isothermal titration calorimetry, MCR, mean count rate, mGCAP2, myristoylated GCAP2, MM, molecular mass, NCS, neuronal calcium sensor, nmGCAP2, nonmyristoylated GCAP2, PDE, phosphodiesterase 6, SEC, size-exclusion chromatography

## Abstract

In murine and bovine photoreceptors, guanylate cyclase–activating protein 2 (GCAP2) activates retinal guanylate cyclases (GCs) at low Ca^2+^ levels, thus contributing to the Ca^2+^/cGMP negative feedback on the cyclase together with its paralog guanylate cyclase–activating protein 1, which has the same function but different Ca^2+^ sensitivity. In humans, a GCAP2 missense mutation (G157R) has been associated with inherited retinal degeneration (IRD) *via* an unknown molecular mechanism. Here, we characterized the biochemical properties of human GCAP2 and the G157R variant, focusing on its dimerization and the Ca^2+^/Mg^2+^-binding processes in the presence or absence of N-terminal myristoylation. We found that human GCAP2 and its bovine/murine orthologs significantly differ in terms of oligomeric properties, cation binding, and GC regulation. Myristoylated GCAP2 endothermically binds up to 3 Mg^2+^ with high affinity and forms a compact dimer that may reversibly dissociate in the presence of Ca^2+^. Conversely, nonmyristoylated GCAP2 does not bind Mg^2+^ over the physiological range and remains as a monomer in the absence of Ca^2+^. Both myristoylated and nonmyristoylated GCAP2 bind Ca^2+^ with high affinity. At odds with guanylate cyclase–activating protein 1 and independently of myristoylation, human GCAP2 does not significantly activate retinal GC1 in a Ca^2+^-dependent fashion. The IRD-associated G157R variant is characterized by a partly misfolded, molten globule-like conformation with reduced affinity for cations and prone to form aggregates, likely mediated by hydrophobic interactions. Our findings suggest that GCAP2 might be mostly implicated in processes other than phototransduction in human photoreceptors and suggest a possible molecular mechanism for G157R-associated IRD.

The phototransduction cascade in vertebrates is finely regulated by the subtle changes in intracellular Ca^2+^, which follow the closure of cyclic nucleotide–gated (CNG) channels upon the light-induced activation of the 3′,5′- cGMP phosphodiesterase 6 (PDE) (1). Guanylate cyclase–activating proteins (GCAPs) are among the neuronal calcium sensors (NCSs) that permit such fine regulation and tune the light sensitivity of rods and cones, thus contributing to shape their photoresponses (1, 2). The widely established function of GCAPs is the regulation of the membrane-bound guanylate cyclases (GCs) *via* a Ca^2+^-dependent feedback mechanism that renders the enzymatic activity dependent of intracellular [Ca^2+^] (3). GCAPs constitutively interact with their GC targets (4, 5, 6) and are able to detect changes in intracellular [Ca^2+^]. In the low intracellular Ca^2+^ phase, when CNG channels are closed because of PDE-mediated cGMP depletion, GCAPs stimulate the production of cGMP. When cGMP levels are sufficiently high, CNG channels reopen, restoring the submicromolar Ca^2+^ levels typical of dark-adapted cells. Finally, GCAPs switch to a Ca^2+^-bound, GC-inhibiting state, bringing the catalytic activity down to or below the basal levels (1, 7).

GCAPs share a high degree of sequence identity, and the available three-dimensional structures of three isoforms (8, 9, 10) call for high structural similarity among the homologs. The general fold is typical of the EF-hand domain pair family, where two globular domains (N- and C-terminal) made of two EF-hand motifs are flanked to each other. The three functional EF-hand motifs permit the binding of up to three cations, either Ca^2+^ or Mg^2+^, in the specific region defined by the helix-loop-helix motif (see Fig. 1*A* for a structural model of guanylate cyclase–activating protein 2 [GCAP2]). Up to three GCAPs isoforms have been discovered in mammal photoreceptors, and six to eight in teleost fish (11), thus raising the question as to the physiological meaning of such apparent redundancy. GCAPs display different affinity for Ca^2+^ (12) and are able to replace Ca^2+^ with Mg^2+^ (13, 14, 15), thus switching between different signaling states as a consequence of specific allosteric mechanisms (16, 17).

**Figure 1:**
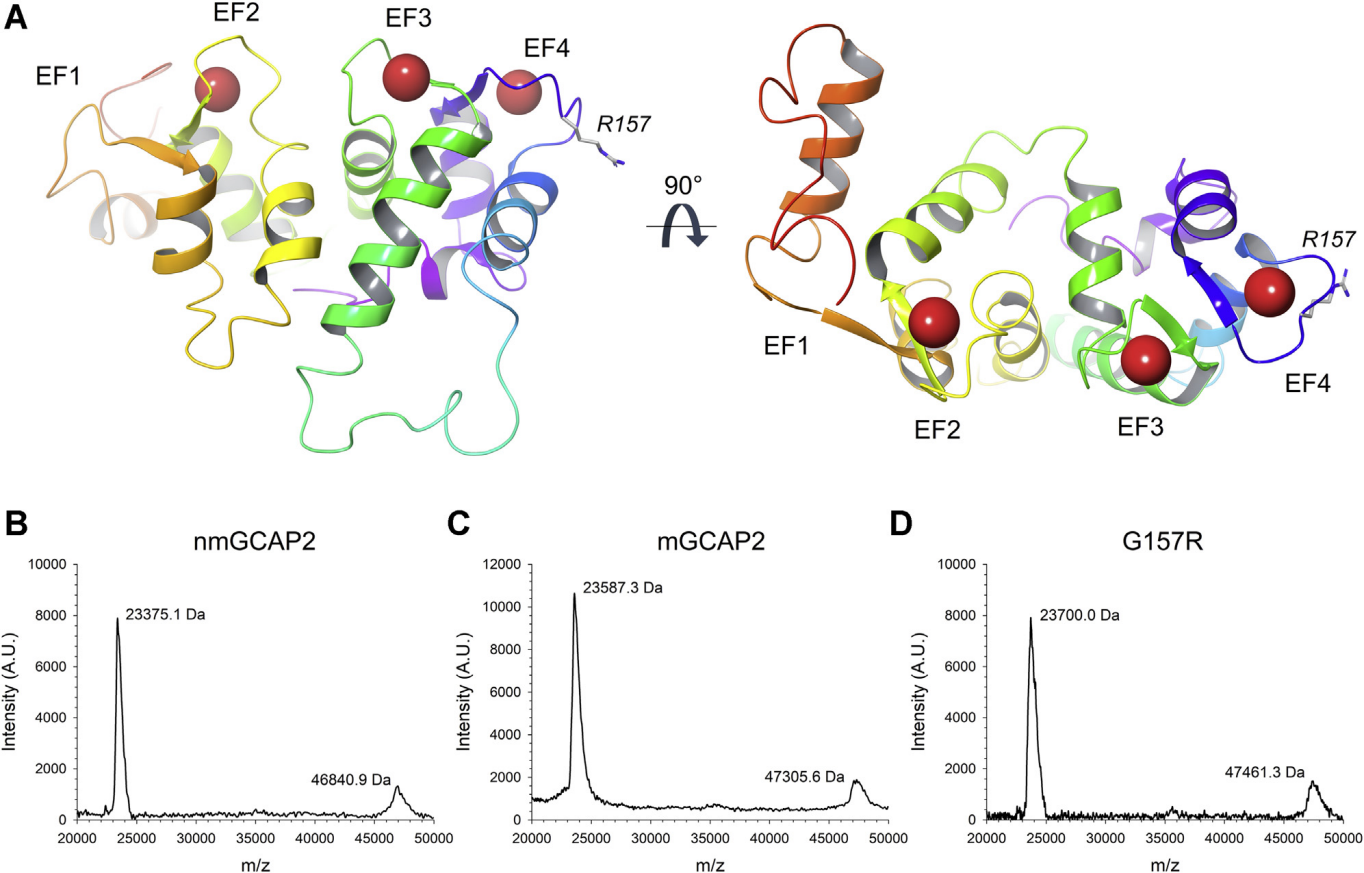
**Three-dimensional structure of nmGCAP2 and assessment of the molecular mass of GCAP2 variants by MALDI-TOF MS.***A*, Cartoon representation of the homology model of human nmGCAP2 (built on bovine nmGCAP2 template, PDB entry 1JBA (8)), colored in a *red-to-purple rainbow* according to the sequence. Ca^2+^ are displayed as *red spheres*, and IRD-associated R157 variant is shown as *white sticks* with N atoms highlighted in *blue*. EF1 to EF4 are labeled, together with residue R157. The protein view is rotated by 90° clockwise along the *x*-axis in the *right panel*. MALDI spectra of (*B*) nmGCAP2, (*C*) mGCAP2, and (*D*) G157R with the molecular mass corresponding to each peak. GCAP2, guanylate cyclase–activating protein 2; IRD, inherited retinal dystrophy; mGCAP2, myristoylated GCAP2; nmGCAP2, nonmyristoylated GCAP2.

The best-known ubiquitous isoforms are guanylate cyclase–activating protein 1 (GCAP1) and GCAP2, which are present both in rods and cones and whose biochemical and physiological features in murine and bovine photoreceptors have been deeply characterized (18, 19, 20, 21, 22). Both GCAP1 and GCAP2 form dimers under physiological conditions (23, 24), and their concerted action is required for the correct Ca^2+^-dependent cGMP synthesis. According to an established relay model in rods (7, 25), GCAP1 has lower affinity for Ca^2+^ and is thus the first GCAP switching to a GC activator as the light-induced drop in calcium levels develops. GCAP2, which has higher affinity for Ca^2+^, activates the GC later in time or at lower Ca^2+^ levels, thus shaping the recovery kinetics of the photoresponse in brighter light or after the peak amplitude under dim light conditions (7). This model has been confirmed by several lines of evidence in bovine (4, 26) and murine studies (22) and is therefore thought to be valid for human photoreceptors. Besides their established activity in the regulation of the phototransduction cascade in murine and bovine photoreceptor outer segments, GCAP1 and GCAP2 have also been localized in the synaptic layer (18, 19), especially suggesting for GCAP2 a Ca^2+^-dependent mediation of the synaptic ribbon plasticity (27).

Although to date more than twenty mutations in the gene *GUCA1A* encoding for GCAP1 have been associated with autosomal dominant cone dystrophy or cone–rod dystrophy (28, 29, 30, 31, 32, 33, 34, 35, 36, 37, 38, 39), only one relatively rare missense mutation substituting Gly157 for an arginine (indicated from now on with G157R) has been found in *GUCA1B* encoding for GCAP2 in Japanese families with a history of retinitis pigmentosa, in some cases with macular involvement (40, 41). The mutation affects a highly conserved residue in the EF4 loop (Fig. 1*A*), compatible with a decrease in the affinity for Ca^2+^ of that binding site. The mutant has been studied *in vivo* by expressing the bovine equivalent to human G157R as a transgene in a mouse model of rods GCAP1/2 KO (42). The study revealed that the mutation caused a significant retention of GCAP2 in the inner segment, which is thought to result in photoreceptor cell death and severe retinal degeneration (42, 43).

To date, no comprehensive biochemical investigation of the molecular properties of human GCAP2 has been performed and no evaluation of the alterations of such properties brought by the G157R variant was available. In this work, we present a thorough characterization of the structural and functional properties of human GCAP2 and its inherited retinal dystrophy (IRD)-associated G157R variant.

## Results

### MS analyses

NCS proteins are subjected to a post-translational modification where a fatty acid (typically myristic acid) is covalently bound to the N-terminal Gly, thus conferring the protein-specific features such as membrane binding or modulation of the Ca^2+^ sensitivity (44, 45). To evaluate the effect of myristoylation on structure–function properties of human GCAP2, we expressed and purified both myristoylated and nonmyristoylated variants and assessed the presence of the post-translational modification by MALDI-TOF MS. The theoretical molecular mass (MM) of nonmyristoylated GCAP2 (nmGCAP2), based on the canonical protein sequence (UniProt entry: Q9UMX6), is reported in Table S1, which also reports the predicted MM of the myristoylated variant (myristoylated GCAP2 [mGCAP2]) both without and with the Gly-to-Arg substitution (G157R). The difference in the main peaks detected by MALDI-TOF spectra for mGCAP2 and nmGCAP2 (212.2 Da) is compatible with the effective myristoylation of the protein. Noticeably, no band ascribable to nmGCAP2 was visible in the mGCAP2 MALDI-TOF spectrum (Fig. 1*B*), thus pointing to a very efficient myristoylation process.

The same analysis performed on the G157R variant showed in a peak at 23.7 kDa (Fig. 1*D*), compatible with a Gly-to-Arg mutation and successful myristoylation, as judged by the substantial correspondence between measured peaks and theoretical values (Table S1). Interestingly, MALDI-TOF spectra showed the presence of dimeric forms in samples of all variants (Fig. 1, *B*–*D* and Table S1).

### Oligomeric state and aggregation propensity of GCAP2 variants

**Figure 2:**
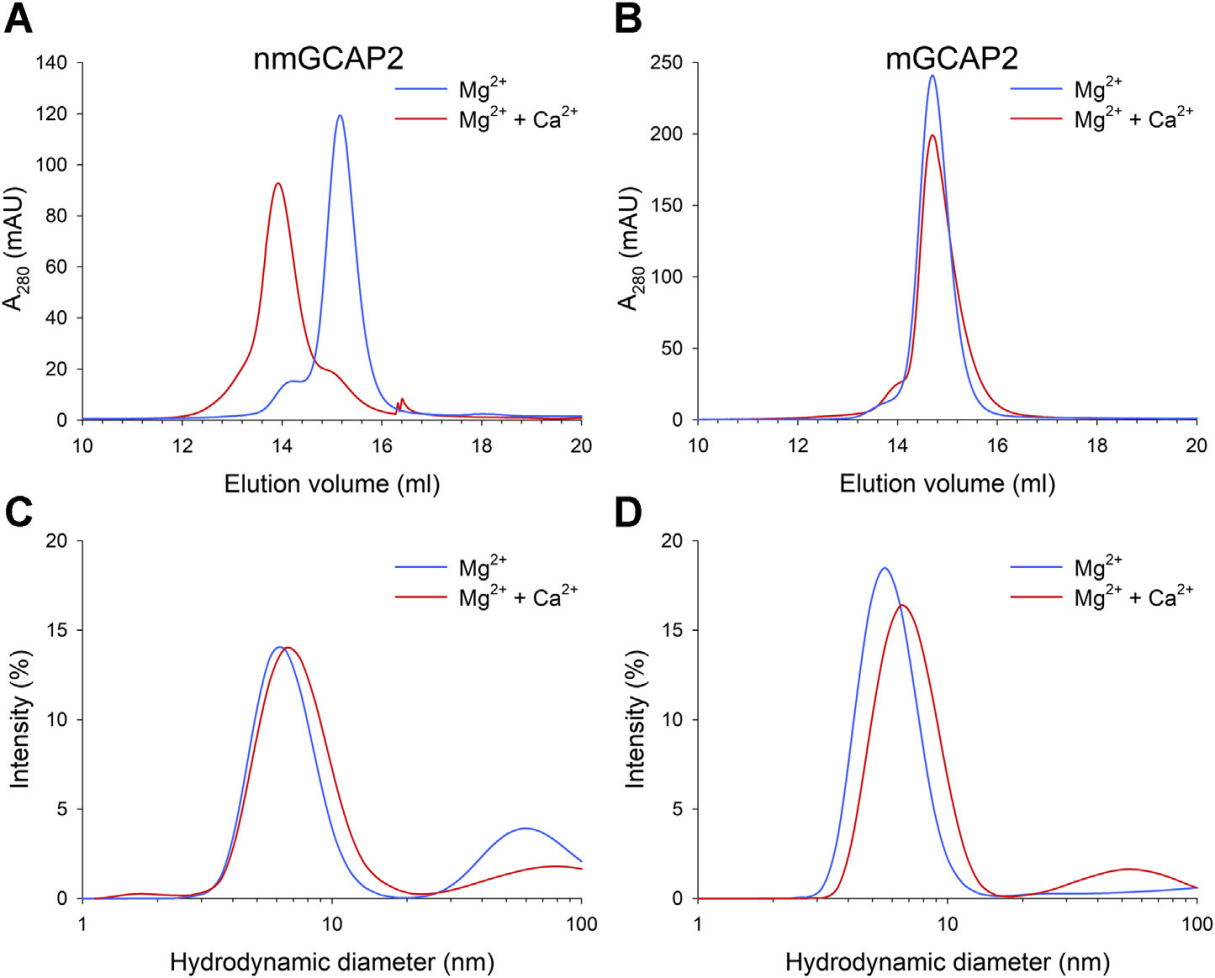
**Effects of Mg**^**2+**^**and Ca**^**2+**^**on the apparent molecular mass and the hydrodynamic diameter of GCAP2 variants.** Analytical SEC profiles of ∼40-μM (*A*) nmGCAP2 and (*B*) mGCAP2 in the presence of 500-μM EGTA + 1-mM Mg^2+^ (*blue*) or 1-mM Mg^2+^ + 500-μM Ca^2+^ (*red*). Hydrodynamic diameter estimation by DLS of ∼40-μM (*C*) nmGCAP2 and (*D*) mGCAP2 in the presence of 500-μM EGTA + 1-mM Mg^2+^ (*blue*) or 1-mM Mg^2+^ + 500-μM Ca^2+^ (*red*). DLS, dynamic light scattering; GCAP2, guanylate cyclase–activating protein; mGCAP2, myristoylated GCAP2; nmGCAP2, nonmyristoylated GCAP2; SEC, size-exclusion chromatography.

NCS proteins are characterized by heterogeneous oligomeric states (23), and it has been reported that the bovine GCAP2 undergoes Ca^2+^-dependent dimerization (46). We investigated whether a similar behavior could be observed for its human ortholog. By performing analytical size-exclusion chromatography (SEC) in the presence of Mg^2+^ or Ca^2+^ on both myristoylated and nonmyristoylated forms, we assessed the effect of cation binding and the post-translational modification on the oligomeric state. The elution profiles of nmGCAP2 showed a main peak at 15.2 ml in the presence of Mg^2+^, which shifted to 14 ml in the concomitant presence of Ca^2+^ (Fig. 2*A*), thus resulting in an estimated MM of 25 and 58 kDa, respectively (see Experimental procedures). On the other hand, SEC profiles of mGCAP2 (Fig. 2*B*) displayed a cation-independent elution peak at 14.7 ml, corresponding to a MM of 35 kDa, which could be related to a different oligomeric state of the protein in Mg^2+^ or Ca^2+^ saturating conditions.

Dynamic light scattering (DLS) conveniently allows measuring the change in the hydrodynamic diameter of calcium sensor proteins resulting from the structural rearrangement upon cation binding, as well as to evaluate any potential time-dependent aggregation propensity by monitoring the mean count rate (MCR) along the time. Results from DLS experiments showed an increase of the hydrodynamic diameter upon Ca^2+^ binding for both GCAP2 forms (Fig. 2, *C* and *D*), although to a different extent. Indeed, nmGCAP2 exhibited a 0.79-nm increase in the hydrodynamic diameter (6.71 *versus* 7.50 nm, Table 1, *p*-value < 0.001), whereas mGCAP2 displayed a 1.06-nm increase upon Ca^2+^ binding (6.02 *versus* 7.08 nm, Table 1, *p*-value < 0.001). Nevertheless, such increase in the hydrodynamic diameter can be ascribable to a conformational change rather than to an aggregation process, as shown by the stability of the MCR over the 90-min experimental time (Fig. S1). Interestingly, in the absence of cations, mGCAP2 displayed an abrupt increase in the MCR and wide oscillations around an approximately 40% higher value compared with the Mg^2+^ or Ca^2+^ saturating conditions, without evidence of aggregation trends (Fig. S1) at odds with nmGCAP2 that, in the apo-form, showed very stable and low MCR values, indicative of a particularly stable colloidal suspension. The same experiments performed with the IRD-associated G157R variant did not allow the estimation of the hydrodynamic diameter because of the high polydispersity index (0.44 ± 0.07 < polydispersity index <0.54 ± 0.08) of the colloidal suspension, indicative of a high tendency to rapidly form aggregates and/or of low protein stability.

**Table 1:** Hydrodynamic diameter estimation of GCAP2 variants

Protein	State	d ± σ (nm) [n]	PdI ± σ
nmGCAP2	Mg^2+^	6.71 ± 0.08 [39]	0.37 ± 0.08
	Mg^2+^ + Ca^2+^	7.50 ± 0.15 [41]	0.31 ± 0.04
mGCAP2	Mg^2+^	6.02 ± 0.06 [55]	0.28 ± 0.04
	Mg^2+^ + Ca^2+^	7.08 ± 0.10 [38]	0.30 ± 0.03

d, hydrodynamic diameter; n, number of measurements; PdI, polydispersity index; σ, standard error.

### Ca^2+^- and Mg^2+^-dependent dimerization of mGCAP2

**Figure 3:**
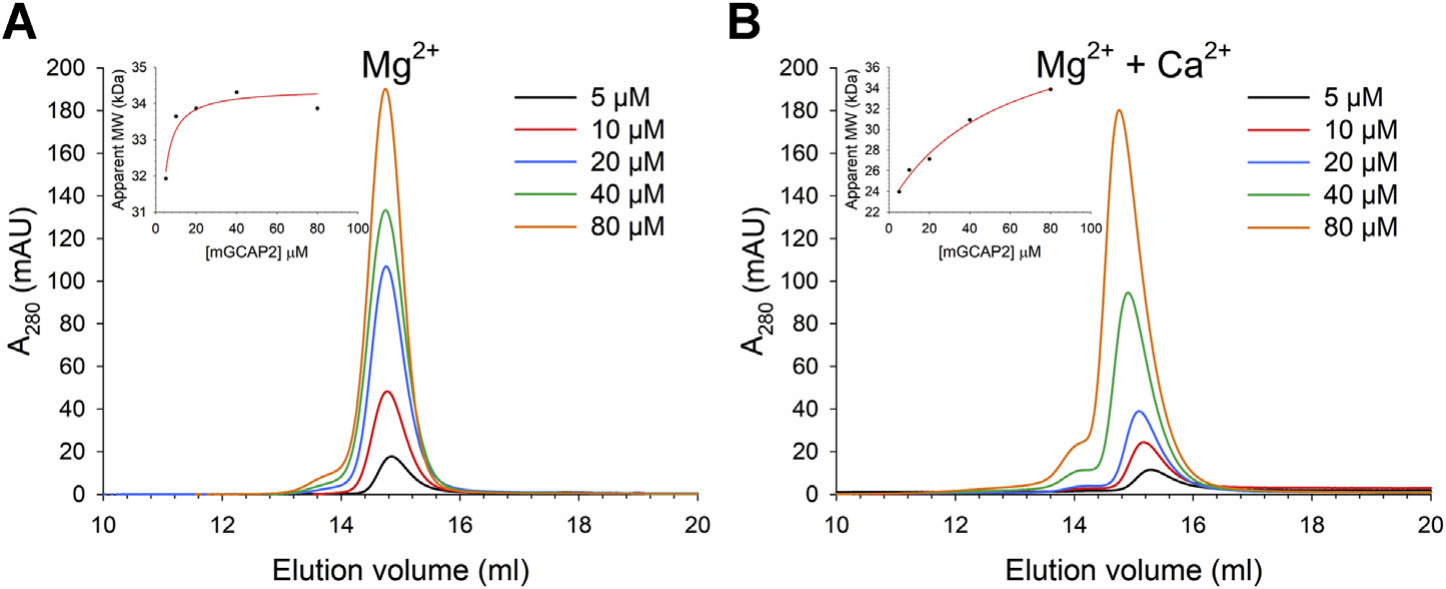
**Effects of Mg**^**2+**^**andCa**^**2+**^**on mGCAP2 dimerization.** Analytical gel-filtration chromatograms of 5-μM (*black*), 10-μM (*red*), 20-μM (*blue*), 40-μM (*green*), and 80-μM (*orange*) mGCAP2 in the presence of (*A*) 500-μM EGTA + 1-mM Mg^2+^ and (*B*) 1-mM Mg^2+^ + 500-μM Ca^2+^. *Insets* show the apparent molecular mass as a function of the mGCAP2 concentration together with the optimal fitting to a hyperbolic curve (see Experimental procedures). GCAP2, guanylate cyclase–activating protein 2; mGCAP2, myristoylated GCAP2.

To investigate the dimerization process of the physiologically relevant myristoylated form of GCAP2 in the presence of cations, the analytical SEC profiles of mGCAP2 were collected at an increasing protein concentration. In the presence of the physiological concentration of Mg^2+^, the elution peak displayed a minor shift of the apparent mass from 31.9 kDa at 5 μM mGCAP2 to 34.3 kDa at concentrations higher than 10 μM (Fig. 3*A*). On the other hand, in the presence of both Mg^2+^ and Ca^2+^, the main elution peak shifted compatibly with an increase of mass from 24 kDa (at 5 μM) to 33.8 kDa at 80-μM mGCAP2 (Fig. 3*B*), moreover, each peak displayed a shoulder at higher MM with the intensity proportional to the protein concentration. By fitting the apparent MM as a function of the mGCAP2 concentration to a hyperbolic curve (Fig. 3, inset), it was possible to obtain a cation-specific apparent *K*_D_ of dimerization. Indeed, in the presence of Mg^2+^ mGCAP2 was found to dimerize with an apparent *K*_D_ < 1 μM (R^2^= 0.89), thus suggesting that with physiological [Mg^2+^] in conditions of very low [Ca^2+^] mGCAP2 is a dimer. On the other hand, in the presence of both Mg^2+^ and Ca^2+^, the estimated apparent *K*_D_ of dimerization shifted to 55.4 μM (R^2^= 0.99), thus pointing toward a Ca^2+^-dependent dimerization of mGCAP2.

The same experiments performed on G157R showed that, despite having the same nominal concentration, all chromatographic profiles (Fig. S2) were considerably lower in intensity than the WT. Such decrease in absorbance pointed toward a massive cation-independent aggregation process, which would render the chromatograms collected at > 20 μM protein almost undetectable, in line with the behavior observed in DLS measurements.

### Stability and conformational changes of GCAP2 variants upon metal cation binding

CD spectroscopy can be used to monitor the conformational changes of NCS proteins upon metal binding, by exploiting the differential absorption of circularly polarized light of the chiral centers of the protein. Specifically, the CD signal in the near UV (250–320 nm) arises from the microenvironment of aromatic residues, thus representing a fingerprint of the protein tertiary structure, whereas in the far UV (200–250 nm), the optically active peptide bonds provide information about the secondary structure elements.

**Figure 4:**
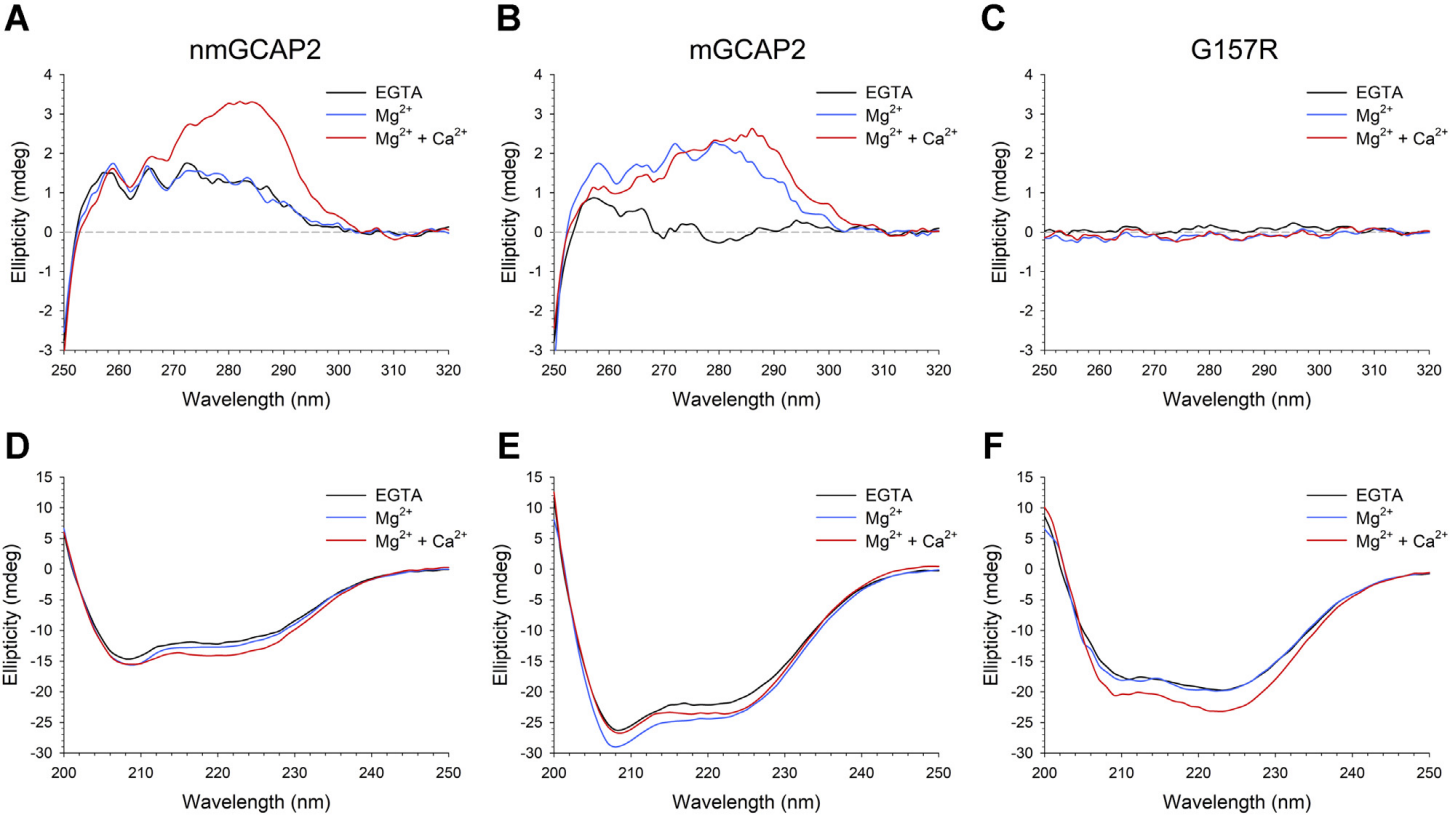
**Conformational changes of GCAP2 variants upon cation binding monitored by CD spectroscopy.** Near-UV CD spectra of 30-μM (*A*) nmGCAP2, (*B*) mGCAP2, and (*C*) G157R in the presence of 500-μM EGTA (*black*) and after addition of 1-mM Mg^2+^ (*blue*) and 1-mM Ca^2+^ (*red*). Far-UV CD spectra of 8-μM (*D*) nmGCAP2, (*E*) mGCAP2, and (*F*) G157R in the presence of 300-μM EGTA (*black*) and after addition of 1-mM Mg^2+^ (*blue*) and 600-μM Ca^2+^ (*red*). GCAP2, guanylate cyclase–activating protein 2; mGCAP2, myristoylated GCAP2; nmGCAP2, nonmyristoylated GCAP2.

The near-UV CD spectrum of apo-nmGCAP2 (Fig. 4*A*, black trace) showed a relatively intense positive signal in all three bands corresponding to aromatic residues (Phe, Tyr, and Trp), indicating that the protein is significantly structured even in the absence of ions. No significant changes in either intensity or fine structure could be observed after the addition of Mg^2+^ (Fig. 4*A*, blue trace), while Ca^2+^ binding resulted in a prominent reshaping of the spectrum, as shown by the increase in ellipticity, particularly in the dichroic bands of Tyr and Trp residues (Fig. 4*A*, red trace). Conversely, apo-mGCAP2 exhibited an almost flat spectrum (Fig. 4*B*, black trace) with a nearly detectable fine structure, while the addition of Mg^2+^ caused a significant increase in ellipticity in all aromatic bands accompanied by a defined fine structure (Fig. 4*B*, blue trace), suggesting that Mg^2+^ is required for the correct folding of mGCAP2. Finally, Ca^2+^ addition resulted in a slight decrease in intensity in the Phe band and a more prominent increase in ellipticity in the Trp band compatible with a small but significant conformational change.

Quite surprisingly, the near-UV CD spectra of the G157R variant were flat under all tested conditions (Fig. 4*C*); moreover, to exclude that this observation could result from the protein being significantly aggregated at the experimental concentration (30 μM), the same experiments were repeated with halved concentration, yielding the same results.

As far as the secondary structure is concerned, all three GCAP2 variants exhibited the typical spectrum of an all α-helix protein (Fig. 4, *D*–*F*), with minima at 208 and 222 nm. The addition of Mg^2+^ to apo-nmGCAP2 resulted in a minor increase in ellipticity at 222 nm (Δθ_222_/θ_222_ = 6.2%, Table 2) with no changes in the spectral shape (θ_222_/θ_208_ = 0.81 in both cases, Table 2). A larger effect was exerted by the addition of Ca^2+^, which resulted in an 18.3% increase in ellipticity at 222 nm accompanied by a significant reshaping of the spectrum (θ_222_/θ_208_ = 0.90, Table 2). On the other hand, mGCAP2 displayed a noticeable increase in CD signal upon Mg^2+^ binding (Δθ_222_/θ_222_ = 10.7%, Table 2) while leaving substantially unaltered the spectral shape (θ_222_/θ_208_ = 0.83 *versus* 0.84, Table 2). Similar to what was observed for nmGCAP2, Ca^2+^ binding affected both the ratio between the typical minima (θ_222_/θ_208_ = 0.89, Table 2) and the intensity, although the increase in ellipticity was smaller than that of nmGCAP2 (Δθ_222_/θ_222_ = 7.8%, Table 2). Surprisingly, the G157R variant exhibited the typical shape (Fig. 4*F*) of a coiled-coil secondary structure in the absence of ions, as shown by θ_222_/θ_208_ = 1.24 (Table 2), which shifted to 1.19 upon addition of Mg^2+^, with negligible effects on the intensity of the spectrum. On the contrary, the addition of Ca^2+^ resulted in a major increase in ellipticity (Δθ_222_/θ_222_ = 18.0%, Table 2), with minor changes in the spectral shape, as θ_222_/θ_208_ = 1.21.

**Table 2:** CD experiment overview

Protein	State	θ_222_/θ_208_	Δθ_222_/θ_222_ (%)	T_m_ (°C)	Unfolding (%)
nmGCAP2	EGTA	0.81		85.0	77.8
	Mg^2+^	0.81	6.2	81.1	80.8
	Mg^2+^ + Ca^2+^	0.90	18.3	>96	36.0
mGCAP2	EGTA	0.83		76.7	82.0
	Mg^2+^	0.84	10.7	76.1	84.9
	Mg^2+^ + Ca^2+^	0.89	7.8	>96	47.5
G157R	EGTA	1.24		67.8	91.6
	Mg^2+^	1.19	1.3	68.7	94.3
	Mg^2+^ + Ca^2+^	1.21	18.0	>96	59.4

Δθ_222_/θ_222_ is calculated as (θ_222_^ion^-θ_222_^EGTA^)/θ_222_^EGTA^. T_m_ is estimated by fitting thermal denaturation profiles to a 4-parameter Hill sigmoid. The percentage of unfolding is calculated as (θ_222_^96^-θ_222_^20^)/θ_222_^20^.

**Figure 5:**
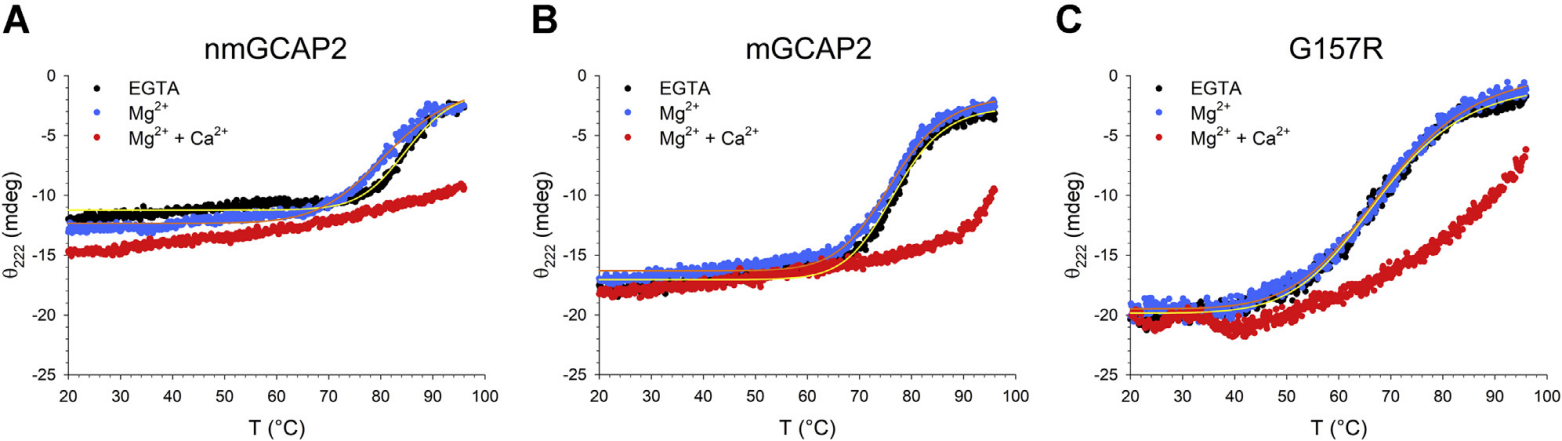
**Cation-dependent thermal stability of GCAP2 variants monitored by CD spectroscopy.** Thermal denaturation profiles of 8-μM (*A*) nmGCAP2, (*B*) mGCAP2, and (*C*) G157R in the presence of 300-μM EGTA (*black*), 300-μM EGTA + 1-mM Mg^2+^ (*blue*) and 1-mM Mg^2+^ + 600-μM Ca^2+^ (*red*). When possible, profiles were fitted to a 4-parameter Hill sigmoid, yielding the T_m_ values reported in Table 2. GCAP2, guanylate cyclase–activating protein 2; mGCAP2, myristoylated GCAP2; nmGCAP2, nonmyristoylated GCAP2.

To assess the thermal stability of the three GCAP2 variants, the protein secondary structure was followed by heating the samples up to 96 °C. The analysis of the thermal denaturation profiles highlighted that Mg^2+^ destabilized nmGCAP2 with respect to the apo-form (ΔT_m_ = −3.9 °C, Table 2), although no clear transition from a folded to an unfolded state could be identified in the presence of Ca^2+^ (Fig. 5*A*). Similarly, the thermal stability of mGCAP2 (Fig. 5*B*) was also slightly decreased in the presence of Mg^2+^ (ΔT_m_ = −0.6 °C, Table 2). It is noteworthy, however, that mGCAP2 was significantly less stable than nmGCAP2 both in the absence (ΔT_m_ = −8.9 °C) and presence of Mg^2+^ (ΔT_m_ = −5 °C). Again, mGCAP2 also did not exhibit complete unfolding upon Ca^2+^ binding up to 96 °C, but a significantly higher percentage of the protein pool was denatured at 96 °C, with respect to nmGCAP2 (unfolding 47.5% *versus* 36%, Table 2). The IRD variant G157R was found to be the least stable in all tested cases (Fig. 5*C*), although the presence of Mg^2+^ slightly increased the thermal stability compared with the apo-form (ΔT_m_ = 0.9 °C). Nonetheless, neither G157R showed a complete unfolding transition in the presence of Ca^2+^ (Fig. 5*C*), although 59.4% of the ellipticity signal was lost at 96 °C (Table 2).

**Figure 6:**
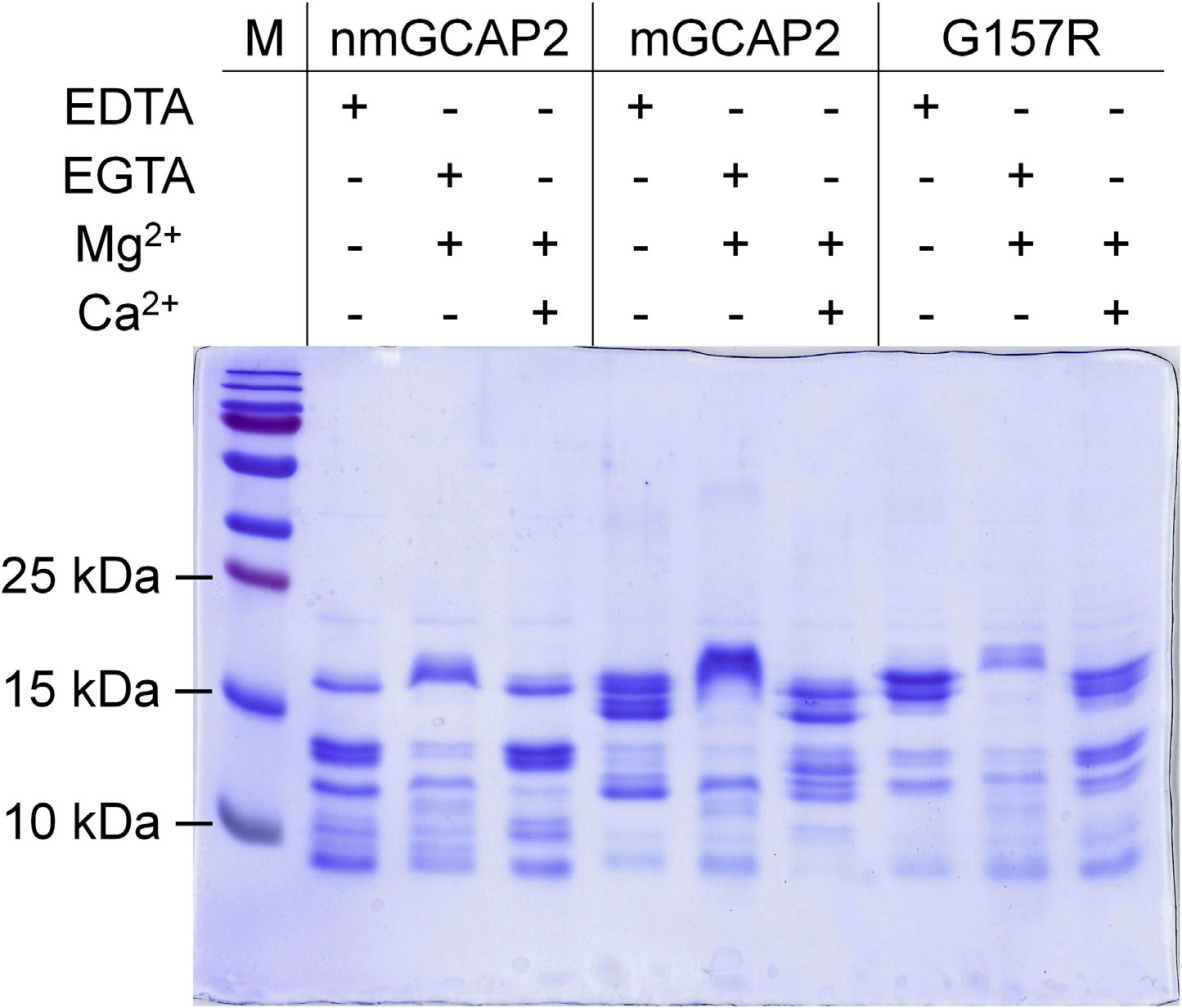
**Conformational changes of GCAP2 variants upon ion binding assessed by proteolytic cleavage.** Limited proteolysis of GCAP2 variants after 10-min incubation with trypsin (60:1 molar ratio) in the presence of 1-mM EDTA, 500-μM EGTA + 1-mM Mg^2+^, or 1-mM Mg^2+^ + 1-mM Ca^2+^. GCAP2, guanylate cyclase–activating protein 2; mGCAP2, myristoylated GCAP2; nmGCAP2, nonmyristoylated GCAP2.

To probe the stability of the GCAP2 variants with a different methodology, we assessed their sensitivity to enzymatic proteolysis (47) in different cation-loading states, which permits a comparative evaluation of conformational and flexibility changes resulting from Mg^2+^ or Ca^2+^ binding (48). We used nmGCAP2 to probe the proteolytic patterns as a function of the incubation time with trypsin (Fig. S3), with complete digestion observed after 30 min of the metal-bound forms. This was at odds with the metal-free form, which presented a native band even after 1 h, indicative of a substantially different conformation of the protein in the apo-state compared to the cation-bound state. To evaluate the cation-dependent proteolytic profiles of all three GCAP2 variants, 10-min incubation time was chosen for comparisons (Fig. 6).

All three variants displayed the residual presence of native protein in the absence of ions (Fig. 6, EDTA lanes) and two major bands at ∼12 and ∼9 kDa. Moreover, nmGCAP2 showed a double band between 12 and 15 kDa, which was less pronounced in the other two variants. In the presence of Mg^2+^, the proteolytic pattern was similar in all cases, with faint bands at lower MM and nmGCAP2 showing a trace of the double band observed in the apo-form, which was absent in the myristoylated case (Fig. 6, Mg^2+^ lanes). Upon Ca^2+^ addition, the native band was again detectable in all GCAP2 variants; moreover, mGCAP2 exhibited a number of proteolytic fragments clustered in the ∼12- to 15-kDa region, while the pattern of both nmGCAP2 and G157 spread over a broader range of MM, with more abundant smaller-sized fragments, especially for the nmGCAP2 case (see bands at around and below 10 kDa). Overall, limited proteolysis experiments confirmed that the effects of Ca^2+^ on GCAP2 conformation and trypsin accessibility depend strongly on peculiar properties of each variant.

### Hydrophobic properties of GCAP2 variants differ upon metal cation binding

GCAPs are hydrophobic molecules (12, 49), a prerequisite for their capability to constitutively bind their GC targets (50). We analyzed how the surface hydrophobicity of the three GCAP2 variants was affected by the presence of metal cations by monitoring changes in the fluorescence emission of 8-anilinonaphthalene-1-sulfonic acid (ANS), which binds to solvent-exposed hydrophobic residues.

**Figure 7:**
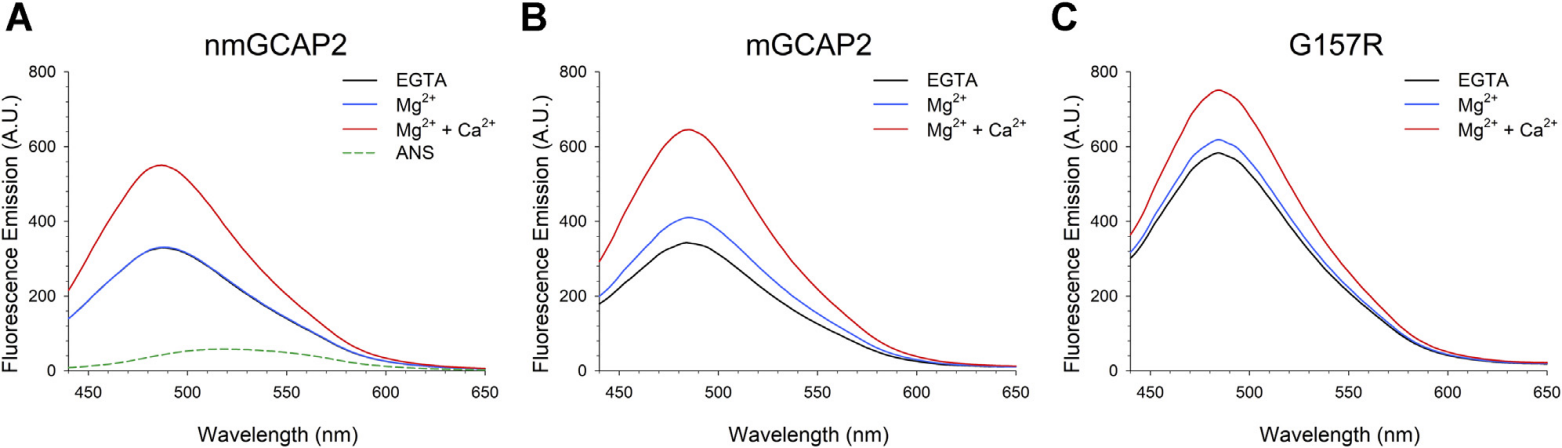
**Variations in hydrophobicity of GCAP2 variants upon cation binding investigated by ANS fluorescence.** Fluorescence spectra of 30-μM ANS and 2-μM (*A*) nmGCAP2, (*B*) mGCAP2, and (*C*) G157R in the presence of 500-μM EGTA (*black*) and after sequential additions of 1-mM Mg^2+^ (*blue*) and 1-mM Ca^2+^ (*red*). The spectrum of ANS is shown as a *green dashed line* in *panel A*. ANS, anilinonaphthalene-1-sulfonic acid; GCAP2, guanylate cyclase–activating protein 2; mGCAP2, myristoylated GCAP2; nmGCAP2, nonmyristoylated GCAP2.

ANS-fluorescence measurements highlighted that all three GCAP2 variants were highly hydrophobic, as shown by the major increase in fluorescence emission and the blue shift exhibited by the ANS probe in the presence of the proteins (Fig. 7). In line with near-UV CD spectra, nmGCAP2 did not show any changes upon Mg^2+^ addition (Fig. 7*A*), either in terms of intensity or shift (ΔI_max_/I_max_ = 0.4%, Table S2) or the wavelength of maximal emission. Ca^2+^ binding to nmGCAP2 was found to expose a larger hydrophobic surface, as ΔI_max_/I_max_ = 67.1% (Table S2). In line with CD spectroscopy and at odds with nmGCAP2, mGCAP2 exhibited sensitivity (Fig. 7*B*) to both Mg^2+^ (ΔI_max_/I_max_ = 19.7%, Table S2) and Ca^2+^ (ΔI_max_/I_max_ = 88.4%, Table S2). It is noteworthy that ΔI_max_/I_max_ for mGCAP2 resulted to be the highest value among the three variants analyzed. The same experiments conducted on G157R suggested that at 2-μM concentration, the IRD-associated variant is significantly more hydrophobic than the WT, both in the absence and in the presence of cations (Fig. 7*C*). Thus, in line with DLS and analytical SEC measurements, these results suggest that the G157R mutant may be susceptible to aggregation mediated by hydrophobic interactions.

### Ca^2+^- and Mg^2+^-binding properties strongly depend on myristoylation

**Figure 8:**
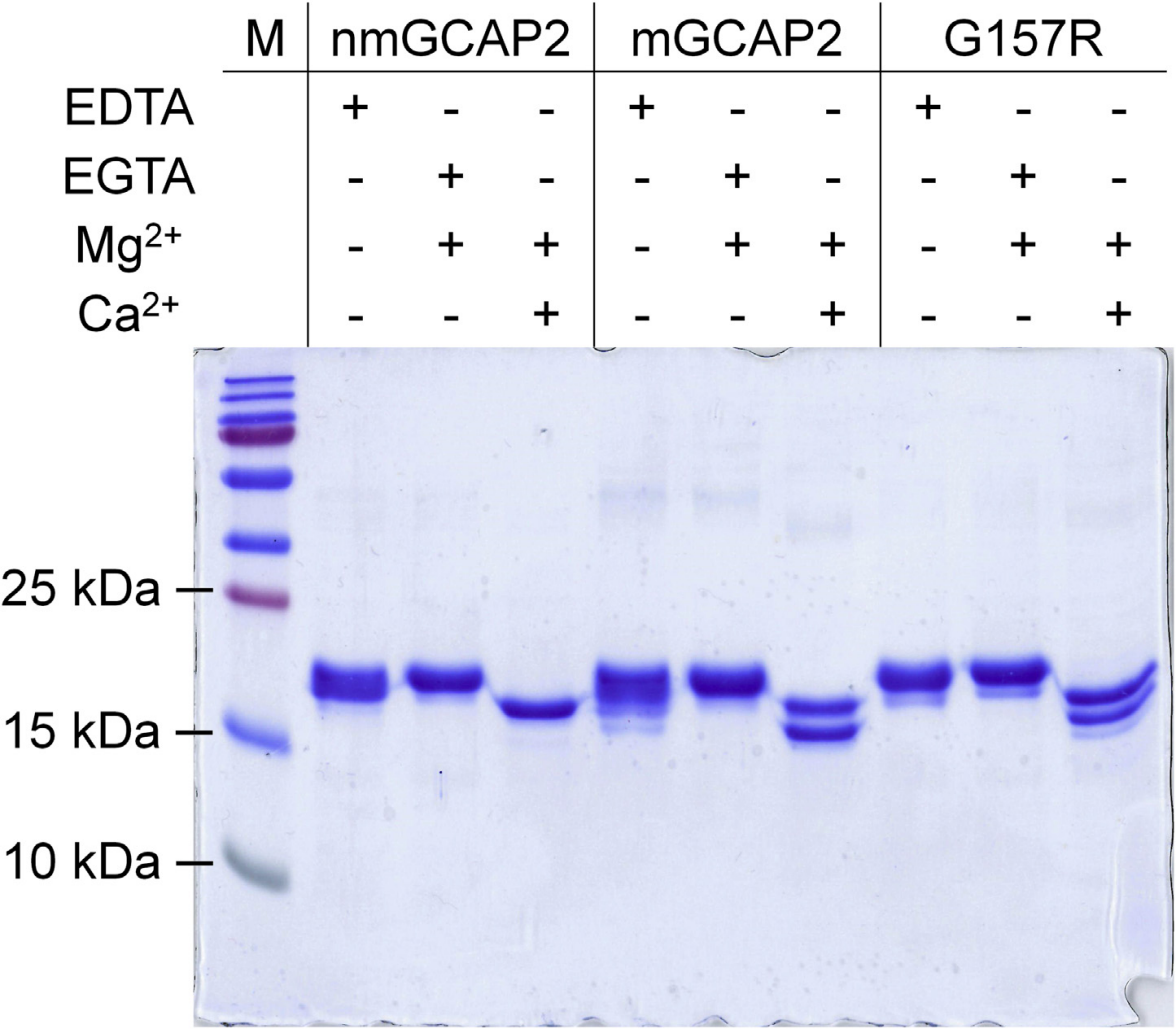
**Effects of Mg**^**2+**^**and Ca**^**2+**^**on the electrophoretic mobility of GCAP2 variants.** SDS-PAGE of 20-μM GCAP2 variants in the presence of 5-mM EDTA, 5-mM EGTA + 1-mM free Mg^2+^, or 1-mM Mg^2+^ + 1-mM Ca^2+^. GCAP2, guanylate cyclase–activating protein 2; mGCAP2, myristoylated GCAP2; nmGCAP2, nonmyristoylated GCAP2.

NCS proteins are known to be subjected to differential electrophoretic mobility in SDS-PAGE experiments under different cation-loading conditions, as their Ca^2+^-bound forms migrate at a lower MM (51). The reason for such phenomenon is not completely understood, but it has been observed that Ca^2+^ sensors change their electrophoretic mobility in a way that is proportional to their Ca^2+^ affinity, with those binding Ca^2+^ more tightly displaying more prominent shifts (12). Interestingly, nmGCAP2 and especially mGCAP2 showed smeared bands in the apo-form, with MM ranging from 23.6 kDa (theoretical mass) to <18 kDa, suggesting high Ca^2+^ affinity (Fig. 8, EDTA lanes), whereas the G157R variant exhibited a more compact smear, pointing toward a reduced affinity for Ca^2+^. The addition of Mg^2+^ led to the presence of a prominent band with a higher apparent MM (Fig. 8, Mg^2+^ lanes), whereas addition of Ca^2+^ confirmed the typical shift to a lower apparent MM of NCS proteins. Indeed, Ca^2+^-bound nmGCAP2 presented a single band at a lower apparent MM, at odds with both WT and G157R mGCAP2, which presented two separate bands.

**Figure 9:**
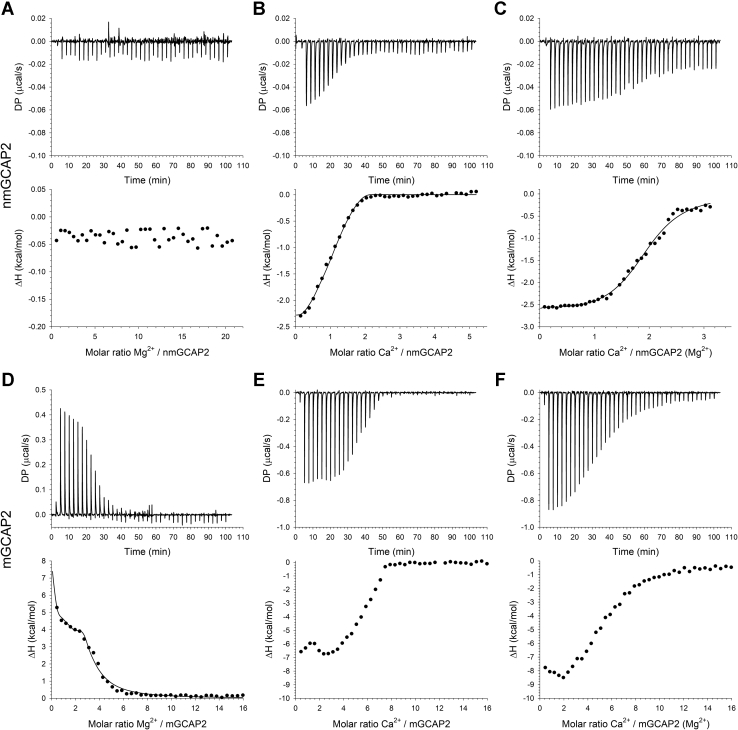
**Metal cations binding to GCAP2 variants measured by ITC.** Representative ITC titrations of 20-μM nmGCAP2 (*A–C*) and mGCAP2 (*D–F*). Mg^2+^ titrations of (*A*) nmGCAP2 and (*D*) mGCAP2. Ca^2+^ titrations of (*B*) nmGCAP2 and (*E*) mGCAP2 in the absence and in the presence (*C*) and (*F*) of 1-mM Mg^2+^. *Upper panels* show heat pulses, and *lower panels* represent molar enthalpy changes relative to each injection. When possible, data were fitted to a two- or three-sequential binding sites (see Experimental procedures), yielding apparent dissociation constants (*K*_D_), enthalpy changes (ΔH), and entropy changes (−TΔS) reported in Tables 3 and 4. GCAP2, guanylate cyclase–activating protein 2; ITC, isothermal titration calorimetry; mGCAP2, myristoylated GCAP2; nmGCAP2, nonmyristoylated GCAP2.

To investigate the thermodynamic properties of Mg^2+^ and Ca^2+^ binding to GCAP2 variants, we performed isothermal titration calorimetry (ITC) measurements by titrating apo-mGCAP2/nmGCAP2 with Mg^2+^ or Ca^2+^. Ca^2+^ titrations were also repeated in the presence of physiological levels (1 mM) of free Mg^2+^. Proteins were carefully decalcified according to a recently optimized protocol (37, 52), which is a crucial step considering the submicromolar affinity of GCAPs for Ca^2+^ (12, 53). Interestingly, when performing Mg^2+^ titrations with apo-nmGCAP2, a basically flat isotherm was observed (Fig. 9*A*). The lack of net change in heat might result from compensating ΔH terms arising from binding and conformational change processes. However, the results obtained with ANS fluorescence (Fig. 7*A*) and near-UV CD (Fig. 4*A*) exclude conformational changes and rather suggest no binding of Mg^2+^ or a high-millimolar affinity binding, which would be undetectable with ITC measurements and of no physiological relevance. On the other hand, Ca^2+^ titrations performed with apo-nmGCAP2 (Fig. 9*B*) showed an exothermic pattern, which could be fitted only to a two-sequential binding site model with affinities in the low and mid nanomolar range (35.2 and 500.5 nM, respectively, Table 3). We cannot exclude that, in spite of the accurate decalcification procedure, the third binding site was already occupied by a Ca^2+^ because of the presence of 80- to 200-nM Ca^2+^ in the decalcified ITC buffer. Ca^2+^ binding to apo-nmGCAP2 was found to be entropy-driven for both resolved binding sites, as shown by the higher contribution of the entropic term −TΔS (−8.1 and −8.6 kcal/mol, Table 3) with respect to the enthalpic term ΔH (−2.1 and −0.05 kcal/mol, Table 3). Interestingly, the same titrations performed in the presence of 1-mM Mg^2+^ (Fig. 9*C*) yielded lower affinities for both resolved binding sites, as the apparent dissociation constants were 3- to 4-fold higher (109.3 *versus* 35.2 nM, and 2.1 *versus* 0.5 μM, Table 3), compatible with an electrostatic screening effect induced by Mg^2+^. Similar to the titrations in the absence of Mg^2+^, Ca^2+^ binding to nmGCAP2 was found to be exothermic and entropy driven, with the main difference being the enthalpically more favorable binding to the second site in the presence of Mg^2+^ (−2.4 kcal/mol *versus* −0.05 kcal/mol, Table 3).

**Table 3:** Thermodynamic parameters for Ca^2+^ binding to nmGCAP2 in the absence (top) and in the presence of 1-mM Mg^2+^ after fitting ITC data to a two sequential binding site model (mean ± SD, n = 3–4)

nmGCAP2 Ca^2+^ titration (2-site model)					
Apparent dissociation constant K_D_		Enthalpy change ΔH (kcal/mol)		Entropy change −TΔS (kcal/mol)	
K_D_^1^ (nM)	K_D_^2^ (nM)	ΔH^1^	ΔH^2^	−TΔS^1^	−TΔS^2^
No Mg^2+^					
35.2 ± 10.4	500.5 ± 273.5	−2.1 ± 0.2	−0.05 ± 0.31	−8.1 ± 0.3	−8.6 ± 0.6
With 1-mM Mg^2+^					
109.3 ± 95.5	2.1 ± 0.6	−2.5 ± 0.2	−2.4 ± 0.5	−7.1 ± 0.7	−5.4 ± 0.6

In stark contrast with nmGCAP2, titrations of mGCAP2 were compatible with a high-affinity Mg^2+^-binding process with an endothermic pattern (Fig. 9*D*). Data could be fit to a three-sequential binding site model yielding apparent dissociation constants ranging from 8.6 nM to 10.2 μM (Table 4), thus suggesting complete saturation under physiological Mg^2+^ levels. Interestingly, binding was found to be endothermic for the two binding sites with the lowest affinity (ΔH = 15.2 and 8.6 kcal/mol, respectively, Table 4), while the highest affinity binding site exhibited a variation in enthalpy close to 0. In addition, Mg^2+^ binding to mGCAP2 was entropy driven for all three binding sites, as was the case for Ca^2+^ binding to nmGCAP2, with entropic contribution spanning between −4.3 and −23.6 kcal/mol.

**Table 4:** Thermodynamic parameters for Mg^2+^ binding to mGCAP2 after fitting ITC data to a three-sequential binding site model (mean ± SD, n = 2)

mGCAP2 Mg^2+^ titration (3-site model)								
Apparent dissociation constant K_D_			Enthalpy change ΔH (kcal/mol)			Entropy change −TΔS (kcal/mol)		
K_D_^1^ (μM)	K_D_^2^ (nM)	K_D_^3^ (μM)	ΔH^1^	ΔH^2^	ΔH^3^	−TΔS^1^	−TΔS^2^	−TΔS^3^
0.72 ± 0.13	8.6 ± 0.7	10.2 ± 1.9	15.20 ± 0.14	0.02 ± 0.76	8.6 ± 0.8	−23.6 ± 0.3	−4.43 ± 0.10	−15.4 ± 0.7

Although Ca^2+^ titrations of mGCAP2 were qualitatively reproducible both in the absence (Fig. 9*E*) and presence of 1-mM Mg^2+^ (Fig. 9*F*), both titration curves showed an inflection point that significantly exceeded the number of functional binding sites of a mGCAP2 monomer (3 potential binding sites). Thus, data fitting to either a two- or three-binding site model yielded no reasonable values for apparent dissociation constant, enthalpy, and entropy changes. A possible explanation of such unusual behavior may rise from the analysis of analytical SEC (Fig. 3*B*) and near-UV CD (Fig. 4*B*) data, which showed that mGCAP2 undergoes both dimerization and conformational changes upon Ca^2+^ binding, therefore making impossible to dissect the different contributions to heat variation. With this notion in mind, ITC data suggest the transition from apo-, monomeric mGCAP2 to Ca^2+^-loaded, dimeric mGCAP2 to be exothermic. Of note, the molar ratio corresponding to the saturation of the process shifted from ∼6(Ca^2+^/mGCAP2) in the absence of Mg^2+^ to >10 in the presence of 1-mM Mg^2+^, suggesting that the high affinity for Mg^2+^ exhibited by mGCAP2 may result in a competition between the two metal cations, thus decreasing its affinity for Ca^2+^.

### GC regulation

The main isozyme of the membrane-bound GC, present both in cones and rods, is GC1 (54) (also referred to as GC-E, ROS-GC1, or RetGC1), and the other GC2 isozyme is producing less than 30% of cGMP in the murine retina (22) and is 25-fold less abundant than GC1 in bovine rod outer segments (55). To assess the potential contribution of GCAP2 to phototransduction in human photoreceptors, we therefore characterized the ability of the GCAP2 variants to activate human GC1 expressed in HEK cells. Our group recently reported that, at odds with bovine and murine orthologs, human mGCAP2 was not able to significantly regulate GC1 activity *in vitro*, in reconstitution experiments that confirmed the capability of human mGCAP1 to regulate the cyclase (56). A similar result was independently observed by Wimberg *et al*. (57) when reconstituting bovine GCAP2 with human GC1. These authors found 8- to 9-fold less activation of human GC1 by GCAP2 than by GCAP1.

To confirm this unexpected result, we performed enzymatic assays, increasing the reaction time with respect to our previous experiments (10 min *versus* 5 min) and assessed the cGMP levels by HPLC (see Experimental procedures). The comparison of mGCAP1- and mGCAP2-mediated regulation of GC1 (Table 5) confirmed our previous results (56). At odds with the behavior of mGCAP1, which was able to activate the target GC1 at low Ca^2+^ (∼34-fold higher than the control) and bring the activation down to basal levels at high Ca^2+^ (*p*-value < 0.001), the activation of GC1 in the presence of mGCAP2 was found to be only ∼3-fold higher than the control and substantially Ca^2+^ independent (Table 5). The maximal activation of GC1 induced by mGCAP2 was not even 12% that of mGCAP1 (Table 5), basically reproducing our previous data (56). The same assays performed with nmGCAP2 showed a Ca^2+^-independent and very mild activation of GC1 (16% compared with mGCAP1). Finally, the G157R variant led to activity levels comparable with those of the controls, thus excluding any relevant activation (Table 5), although the regulation was Ca^2+^ dependent (*p*-value < 0.001).

**Table 5:** Comparison of GC1 regulation by GCAP1 and GCAP2 variants

Protein	cGMP production (pmol/min) [n]	
	Low Ca^2+^	High Ca^2+^
Control	1.14 ± 0.05 [2]	1.12 ± 0.04 [2]
mGCAP1	39.5 ± 3.8 [3]	2.0 ± 0.5 [3]
mGCAP2	4.6 ± 0.2 [2]	4.3 ± 0.4 [2]
nmGCAP2	6.2 ± 0.6 [3]	6.0 ± 0.5 [3]
G157R	1.3 ± 0.1 [3]	0.68 ± 0.03 [3]

Data are reported as mean ± SD, and n represents the number of independent technical replicas.

## Discussion

### Myristoylation modulates the oligomeric state and the Mg^2+^/Ca^2+^ sensitivity of human GCAP2

Myristoylation has been shown to significantly affect bovine GCAP1 function by modulating the regulation of GC1 (58) as well as Ca^2+^ affinity and protein stability (53). Myristoylation was also found to influence GCAP1 intracellular location, as nmGCAP1 was retained in the inner segment of murine photoreceptors (42). Conversely, myristoylation of bovine GCAP2 significantly affected neither the ability to regulate GC1 nor the catalytic efficiency of the enzyme (4, 58).

We previously showed that myristoylation of bovine GCAP2 may affect its structural properties in a rather complex manner (17). Indeed, in spite of the extremely high sequence identity (84.5%), some apparent differences become obvious when comparing the structural response to cations of bovine and human GCAP2. Although near-UV CD spectra of bovine mGCAP2 and nmGCAP2 were similarly affected by the presence of Mg^2+^ (17), the human ortholog showed a significant structural response only in the case of mGCAP2 (Fig. 4, *B* and *E*), in line with the high-affinity binding demonstrated also by ITC (Fig. 9 and Table 3) and ANS fluorescence (Fig. 7). Indeed, human mGCAP2 displayed a way more prominent response to Mg^2+^ starting from the apo-form (Fig. 4, *B*–*E*), which appeared essentially less structured compared with the nmGCAP2 variant. The latter had already a more defined tertiary structure in the absence of cations (Fig. 4, *A*–*D*) but did not show significant Mg^2+^-induced conformational change.

Another important discrepancy between bovine and human orthologs regards the thermodynamic characteristics of Mg^2+^ binding to nmGCAP2 and mGCAP2. In line with the present study (Fig. 9*A*), for bovine nmGCAP2, no ITC pattern compatible with Mg^2+^ binding was observed starting from the apo-form. However, variable and puzzling results were observed for bovine mGCAP2 (17) because only less than half of the ITC experiments showed patterns compatible with binding, and when observed, the patterns were exothermic (17). This is at odds with current data on human mGCAP2, which showed highly reproducible endothermic Mg^2+^ binding with a 3:1 cation:protein stoichiometry and affinities compatible with full saturation in cellular conditions (Fig. 9, Table 4). Therefore, a subtle link between myristoylation and response to Mg^2+^ exists for human GCAP2, but not for the bovine ortholog.

Bovine mGCAP2 was previously shown to undergo Ca^2+^-dependent dimerization (46) and a more recent study using sedimentation equilibrium estimated a *K*_D_ = 52 μM for the monomer/dimer equilibrium in the presence of saturating Ca^2+^ (24). To our knowledge, neither of those studies focused on the potential role of Mg^2+^ and myristic moiety on the dimerization process. We investigated this aspect in the present study and found that the myristic moiety is crucial for determining the oligomeric state of human GCAP2. Indeed, in the absence of Ca^2+^ and in the presence of physiological levels of Mg^2+^, nmGCAP2 was monomeric (Fig. 2*A*), but in the same conditions, the myristoylated form promptly shifted to a dimer (Fig. 1*B*). Therefore, mGCAP2 present in the photoreceptor outer segment under conditions of very low Ca^2+^ corresponding to intense illumination would likely be a dimer (Fig. 3*A*). However, under Ca^2+^-saturating conditions, such as those typical of dark-adapted cells, a clear equilibrium between monomeric and dimeric forms was observed (Fig. 3*B*). Our analytical SEC data quantitatively match the finding in Pettelkau *et al.* (24) that GCAP2 forms dimers under saturating Ca^2+^ conditions (*K*_D_ = 55.4 μM), and this evidently occurs also in the copresence of Mg^2+^. While the copresence of monomers and dimers is in principle possible for all the tested variants, as highlighted by MALDI-TOF measurements (Fig. 1*B*), a complex relationship between cation levels and myristoylation evidently determines the actual oligomeric state of human GCAP2 in specific signaling conditions.

Thermal denaturation studies suggest that Mg^2+^ slightly destabilizes mGCAP2 (Table 2), but the destabilization is more significant for nmGCAP2 (Table 2). Instead, Ca^2+^ binding stabilized all the GCAP2 variants, but the lowest degree of thermal unfolding was observed for nmGCAP2 (Table 2), which apparently does not bind Mg^2+^ as confirmed by ITC and CD spectroscopy (Figs. 9*A* and 4*A*). Analysis of the proteolytic patterns for nmGCAP2 (Fig. S3) seems to apparently contradict thermal denaturation profiles, as under the tested conditions, the apo-form was the one less prone to protease digestion, whereas in conditions of saturating Mg^2+^ or Ca^2+^, the trypsin cleavage was completed rather soon. However, the contradiction is only apparent, as the proteolytic method is only indirectly related to stability, as it is rather sensitive to protein conformation. Indeed, nmGCAP2 was clearly already structured in the absence of cations (Fig. 4*A*), in contrast to apo-mGCAP2, which appeared to show a much lower degree of tertiary structure (Fig. 4*B*) also confirmed by the oscillations of the hydrodynamic diameter observed in time-resolved DLS (Fig. S1*B*) pointing to a dynamic molten globule state rather than to an aggregation-prone conformation.

The specificity and peculiarity of the signaling states of m- and nm-human GCAP2 is reflected by the hydrodynamic size of the two variants, which increased upon addition of Ca^2+^ to a different extent (Table 1, Fig. 2, *C* and *D*) indicative of specific hydrodynamic and hydrophobic properties also revealed by ANS fluorescence (Fig. 6). Both variants indeed bound Ca^2+^ on top of Mg^2+^, although with specific patterns (Fig. 9). It is extremely difficult to estimate reliable binding constants for mGCAP2 by ITC, likely due to the concomitant dimerization and conformational change processes whose specific enthalpic contribution cannot be dissected. A similar phenomenon was already observed for bovine mGCAP2 (17). Probably, only high-resolution analytical techniques such as titrations performed by NMR could help solving individual equilibria, thus providing robust *K*_D_ estimates for Ca^2+^ binding to each site.

In conclusion, our characterization points to myristoyl acting as a dynamic regulator of human GCAP2 structure and oligomeric state, which allows more prominent and sensitive structural response to Mg^2+^ and Ca^2+^ stimuli. Structural information on bovine GCAP2 showed that, at odds with GCAP1, the myristic moiety could be rather exposed to the solvent in both apo-state and Ca^2+^-bound state (59). This is proved by its noticeable sensitivity to the external environment, as confirmed by experiments with membranes and micelles (60). Our data are substantially in line with this view and further underline other discussed peculiarities of human GCAP2 compared with the bovine ortholog.

### The IRD-associated G157R variant is misfolded and tends to form aggregates independently of Mg^2+^ and Ca^2+^

Our *in vitro* data show that the IRD-associated G157R variant forms a molten globule state deprived of tertiary structure (Fig. 4*C*), yet clearly conserving elements of secondary structure (Fig. 4*F*). Surprisingly, the CD spectra showed features of a coiled-coil assembly that, although less than the WT, responded to Mg^2+^ and Ca^2+^ stimuli (Fig. 4*F*), as confirmed by gel shift assays (Fig. 8) and ANS fluorescence (Fig. 7*C*). G157R was significantly less stable than the WT under all tested conditions (Table 2), and a noteworthy tendency to form aggregates was observed independent of the cations. ANS fluorescence spectra suggest the process to be triggered by the high exposition of hydrophobic patches (Fig. 7*C*).

The subcellular distribution of GCAP2 in murine photoreceptors was shown to strongly depend on its phosphorylation state, with the maximal distribution to the rod outer segment estimated to be approximately 50% (61). According to murine *in vivo* studies, a ∼50% phosphorylation of residue S201 in bovine GCAP2 (S197 in human GCAP2) causes protein sequestration at the inner segment, and the accumulation of GCAP2 variants unable to bind cations in the inner segment induced severe toxicity (61). The equivalent of G157R for bovine GCAP2 (G161R), expressed as a transgene in mice lacking GCAPs, caused the retention of 80% GCAP2 in the inner segment in more than half of the tested cells (42). The mutation also altered the Ca^2+^ dependence of S201 phosphorylation by PKG *in vitro* over Ca^2+^ levels that mimic those in photoreceptors (42). Taken together, our data suggest a parallel between the pathological mechanism in murine and human retina, and support the hypothesis that a poorly defined tertiary structure and reduced ability to respond to Ca^2+^ may enhance the phosphorylation of the G157R variant, which might lead to its accumulation and possibly aggregation in the inner segment, as observed in mouse photoreceptors (42).

### Is human GCAP2 mainly involved in the phototransduction cascade?

Based on robust mathematical models of phototransduction cascade in amphibian (62) and mouse rods (63), we have previously proposed that, in the presence of high expression levels of cone dystrophy–associated GCAP1 mutants blocking GC1 in a constitutively active state, GCAP2 could compensate by dynamically regulating the cGMP synthesis (64). The experimental confirmation of this hypothesis has been recently reported *in vivo* in a mouse model where GCAP2 was shown to contribute to GC1 activation in cones in the absence of GCAP1 (65). Nonetheless, the minor activation of GC1 by mGCAP2 and its independence on Ca^2+^ (Table 5) observed in the present and previous findings (56) do not support a similar hypothesis for human photoreceptors. Indeed, it has been demonstrated *in vitro* that the presence of human GCAP2 could not mitigate the dominant effect of the cone–rod dystrophy–associated E111V-GCAP1 variant leading to abnormally high synthesis of cGMP at physiological levels of Ca^2+^, whereas extra delivery of WT GCAP1 could partly restore the normal cyclase regulation (56). The data presented in this work are fully in line with such previous observations and partly in contrast with those reported in a recent contribution (66) where human mGCAP2 was shown to activate human GC1 to approximately half levels compared with GCAP1. Using an *in vitro* assay similar to the one presented here, the authors interpreted the lack of GCAP2-mediated activation of GC1 reported in our previous work (56) as probably due to a deficiency in the preparation of functional GCAP2 (66). We clearly demonstrate in this study that this is not the case, as recombinant human GCAP2 indeed displays all the biochemical features of a perfectly functioning NCS protein switching between different states, moreover recombinant GCAP1, expressed and purified by a very similar procedure, was fully capable of activating GC1 (Table 5). Our present data show that human GCAP2 is a very weak activator of human GC1 because the observed activity only slightly exceeds that of the control, as observed previously with bovine GCAP2 (57). We do not have an explanation for the discrepancy between our findings and those in Peshenko *et al.* (66), but it should be noted that no investigation of the Ca^2+^ dependence of GC1 activation by GCAP2 was reported in that study; therefore, no conclusion as to the effective dynamic regulation is currently possible. Moreover, in the same study, the reported EC_50_ value (19 μM) for GCAP2-mediated GC1 activation as a function of the protein concentration was approximately 17-fold higher than that observed for human GCAP1 (1.13 μM), indicating a rather low affinity for the human GCAP2–GC1 complex. If the cellular concentration observed in bovine rods (approximately 3 μM for both GCAP1 and GCAP2) is taken as a reasonable reference also for human rods, this would exclude any significant role of human GCAP2 in regulating human GC1 also with the data presented in Peshenko *et al.* (66), at least in the concomitant presence of GCAP1. Considering the neat prevalence of GC1 isozyme as the cyclase involved in the phototransduction cascade in many species, our data do not support a prominent role for GCAP2 in regulating phototransduction in human photoreceptors.

### Alternative roles for human GCAP2

The role of GC2 in human photoreceptors' outer segments is still unknown, and a GCAP2-mediated activation of GC2 similar to the one observed in bovine (4, 13) and mice rods (22, 25, 67) is still to be proven. It is therefore reasonable to expect a minor, if any, role of GCAP2 in regulating phototransduction in human. The possibility that GCAP2 is involved in biological functions not occurring in the outer segment and therefore different from the phototransduction cascade indeed dates back to its discovery (20), mostly supported by the apparent absence of GCAP2 in photoreceptor outer segments in early reports. A thorough study corroborated by electron microscopic analysis localized GCAP2 in monkey and human retinas in both rods and cones, with a higher localization in inner segments than outer segments (68). The pattern of retinal expression of GCAP2 therefore seems to be different between mice and humans (68), and the exerted biological role could therefore be distinct in different species. It is also noteworthy that in various species significant immunoreactivity was detected for GCAP2, but not GCAP1, in bipolar, amacrine, and ganglion cells, while both GCAPs were detected in the synaptic region of the photoreceptors, suggesting again an involvement in pathways other than regulation of phototransduction (69).

Results from *in vivo* studies in mice indicate that GCAP1:GCAP2 relative levels should be preserved for maintaining the integrity of the synaptic terminal (43) and points to a specific role of GCAP2 in remodeling morphological changes of synaptic ribbons. Such process could be mediated by GCAP2 binding to RIBEYE, a component of the synaptic ribbon (27), and could confer a Ca^2+^-dependent plasticity to synaptic processes that would not necessarily require outer segment cyclases as a target. These hypotheses should be specifically tested in future studies, which should also investigate whether and to what extent can human GCAP2 activate human GC2.

## Experimental procedures

### Expression and purification of human WT and G157R GCAP2

The cDNA of human GCAP2 (UniProt entry: Q9UMX6) was cloned into pET-11a(+) vector using *Ndel* and *NheI* as restriction sites (GenScript). The G157R point mutation was introduced by site-directed mutagenesis using QuikChange II Kit (Agilent), and primers (forward: 5′-CAGCTGGCCGTCACGGTTCTCATCAACCA-3′, reverse: 5′-TGGTTGATGAGAACCGTGACGGCCAGCTG-3′) and DNA sequencing were obtained by Eurofins. Nonmyristoylated WT GCAP2 was heterologously expressed in *Escherichis coli* BL21 DE3 cells after transformation with pET-11a(+)-GCAP2 plasmid. Myristoylated WT and G157R GCAP2 were heterologously expressed in *E. coli* BL21 DE3 cells after cotransformation with pET-11a(+)-GCAP2 or pET-11a(+)-GCAP2–G157R and pBB131–yNMT (encoding for N-myristoyl transferase from *S. cerevisiae*) plasmids. The expression and purification protocol for GCAP2 variants was essentially the same as reported in refs. (4, 70). Briefly, bacteria were grown at 37 °C until absorbance at 600 nm reached 0.7, when protein expression was induced with 1-mM IPTG, and finally, the bacterial pellet was collected after 4 h at 37 °C. For myristoylated forms, 50 μg/ml myristic acid (in 50% ethanol, pH 7.5) was added at absorbance at 600 nm = 0.4. After cell lysis, solubilization of inclusion bodies in 6 M guanidinium hydrochloride, refolding by dialysis and ammonium sulfate precipitation, GCAP2 variants were purified using a combination of SEC (HiPrep 26/60 Sephacryl S-200 HR, GE Healthcare) and anion-exchange chromatography (HiPrep Q HP 16/10, GE Healthcare) as described in Marino *et al*. (33), with the only difference being the pH for anion-exchange chromatography buffers (8 for WT, 8.5 for G157R variant). The protein concentration was measured by the Bradford assay (71), using a calibration curve obtained specifically for human GCAP2 after quantification by amino acid hydrolysis analysis (Alphalyse). Protein purity was assessed by SDS-PAGE, and then GCAP2 samples were exchanged in 20-mM Tris HCl, pH 7.5, 150-mM KCl, and 1-mM DTT buffer, flash-frozen, and stored at −80 °C until use.

### MALDI-TOF MS

GCAP2 samples for MALDI-TOF-MS measurements (1.0 mg/ml in 20 mM Tris HCl pH 7.5, 150 mM KCl, 1 mM DTT buffer) were desalted by ZipTip C4 (Millipore), and desalted protein was eluted with 30% (v/v) acetonitrile/0.1% (v/v) formic acid. First, a sinapinic acid matrix layer was prepared by depositing a droplet (0.5 μl) of saturated solution of sinapinic acid in 100% ethanol on the target. Thereafter, the eluted sample was deposited on an MALDI target plate. MALDI analyses were performed with ultrafleXtreme MALDI-TOF/TOF MS provided by the "Centro Piattaforme Tecnologiche" of the University of Verona.

### Analytical SEC experiments

The apparent MM of GCAP2 variants was determined by analytical SEC using a Superose 12 10/300GL (GE Healthcare) column equilibrated with 20-mM Tris HCl, pH 7.5, 150-mM KCl, 1-mM DTT buffer, and 500-μM EGTA + 1-mM Mg^2+^ or 500-μM Ca^2+^ + 1-mM Mg^2+^.

A calibration curve was obtained as described earlier (72). Standard proteins for calibration were carbonic anhydrase (29 kDa), alcohol dehydrogenase (150 KDa), β-amylase (200 kDa), and cytochrome c (12.4 kDa). The elution profiles at λ = 280 nm of ∼40 μM GCAP2 (or in the 5- to 80-μM range for analytical SEC titrations) were recorded at 25 °C to identify the elution volume (V_e_) used to calculate the partition coefficient (K_av_) according to the following equation:

$$K_{av}=\left(V_{e}-V_{v}\right)/\left(V_{t}-V_{v}\right)$$

where V_v_ is the void volume (8 ml) and V_t_ is the total column volume (25 ml). The MM was then extrapolated from the calibration curve of log(MM) as a function of K_av_ described in (72). The apparent dimerization constant was obtained by fitting the MM as a function of the protein concentration to a singular rectangular three-parameter hyperbola:

$$MM=MM_{0}+\frac{a*\left[GCAP2\right]}{b+\left[GCAP2\right]}$$

where *MM*_*0*_ represents the Y-intercept, *a* represents the asymptotic value for MM minus the Y-intercept, and b is the apparent *K*_D_ value obtained by fitting.

### DLS measurements

The hydrodynamic diameter and the time evolution of the MCR of GCAP2 variants were measured with a Zetasizer Nano-S (Malvern instruments) at 25 °C, using the experimental setup detailed in (73). Protein samples were diluted to ∼40 μM in 20-mM Tris HCl, pH 7.5, 150-mM KCl, 1-mM DTT buffer with 500-mM EGTA, with further addition of 1-mM Mg^2+^ or 1-mM Mg^2+^ + 500 μM Ca^2+^ and filtered through a 20-nm cut-off Anotop 10 filter (Whatman) before DLS measurements. Samples were equilibrated at 25 °C for 2 minutes, and 38 to 55 measurements were collected approximately every 2 minutes for >90 min, each consisting of 16 to 18 repetitions.

### CD spectroscopy and thermal denaturation profiles

CD data were collected at 37 °C on a Peltier thermostated Jasco J-710 spectropolarimeter using the same parameters as described previously (33). The spectrum of the buffer (20-mM Tris HCl, pH 7.5, 150-mM KCl, 1-mM DTT) was recorded and subtracted to both near- and far-UV CD spectra. Near-UV CD spectra of 30-μM GCAP2 variants were collected between 250 and 320 nm in 1-cm quartz cuvette in the presence of 500-μM EGTA, and after sequential additions of 1-mM Mg^2+^ and 1-mM Ca^2+^, leading to approximately 500-μM free Ca^2+^. Far-UV CD spectra of 8-μM GCAP2 variants were collected in the 200- to 250-nm range in 1-mm quartz cuvettes in the presence of 300-μM EGTA and after sequential additions of 1-mM Mg^2+^ and 600-μM Ca^2+^, leading to approximately 300-μM free Ca^2+^.

The thermal denaturation profiles were recorded in the same experimental conditions as far-UV CD spectra on the same samples using the following parameters: temperature range 20 to 96 °C, scan rate 1 °C/min, response time 4 s, and wavelength 222 nm. Assuming a two-state transition process where the ellipticity signal as a function of the temperature represents the fraction of folded/unfolded protein, thermal denaturation profiles were fitted to a following 4-parameter Hill sigmoid:

$$y=y_{0}+\;\frac{ax^{b}}{\left(c^{b}+x^{b}\right)}$$

Where *y*_*0*_ is the ellipticity value at 20 °C, *a* is the variation in ellipticity between 96 °C and 20 °C, *b* is the Hill coefficient, *c* is the inflection point of the sigmoid, which represents the melting temperature.

### SDS-PAGE mobility shift assays

A 15% acrylamide SDS-PAGE was performed to evaluate the cation-induced differential electrophoretic mobility of GCAP2 variants under denaturing conditions. Proteins were diluted in 20-mM Tris HCl, pH 7.5, 150-mM KCl, and 1-mM DTT buffer to 20 μM and loaded on the gel after 5 min incubation with 5-mM EDTA, 5-mM EGTA +1-mM Mg^2+^ or 1-mM Mg^2+^ + 5-mM Ca^2+^.

### Limited proteolysis

Limited proteolysis of 25-μM GCAP2 variants in the presence of 1-mM EDTA, 500-μM EGTA +1-mM Mg^2+^ or 1-mM Mg^2+^ + 1-mM Ca^2+^ was performed after incubation at 25 °C with trypsin in an enzyme:protein molar ratio of 1:60. Enzymatic cleavage was stopped after 5, 10, 15, 30, 45, 60, and 90 min through heat inactivation of trypsin, and then, samples were loaded onto a 15% SDS gel (Fig. S3). Proteolytic patterns reported in Figure 6 refer to 10-min incubation with trypsin.

### ITC

Cation binding to GCAP2 variants was monitored by ITC using a MicroCal PEAQ-ITC (Malvern Instruments) following the decalcification procedure detailed previously in refs. (37, 52). Briefly, protein samples were dialyzed overnight against 20-mM Hepes, pH 7.4, 60-mM KCl, 4-mM NaCl buffer, and then buffer and samples were decalcified before ITC measurements with a gravity-packed Chelex 100 (Bio-Rad) column, resulting in a final free Ca^2+^ of 80 to 200 nM. Titrations of 8- to 20-μM nmGCAP2 were performed at 25 °C by 40 consecutive injections of 1 μl of 2- to 7-mM Mg^2+^ or 150-μM – 1-mM Ca^2+^ in the absence and in the presence of 1-mM Mg^2+^. Titrations of 13- to 27-μM mGCAP2 were performed at 25 °C by injecting 1 μl of 1- to 2-mM Mg^2+^ or 1- to 2-mM Ca^2+^ in the absence and in the presence of 1-mM Mg^2+^. Injections scheme for each experiment consisted of 150- or 210-s initial delay and 180-s injection space. For each condition, a minimum of two independent technical repetitions were obtained and subjected to data fitting after adjusting the baseline using the fitting offset criterion, thus excluding enthalpic contributions arising from the dilution of cations into buffer. Ca^2+^ titrations of nmGCAP2 were fitted by MicroCal PEAQ-ITC Analysis Software (Malvern Instruments) to a two-sequential binding site model, although no binding was identified in Mg^2+^ titrations. Mg^2+^ titrations of mGCAP2 were fitted by MicroCal PEAQ-ITC Analysis Software (Malvern Instruments) to a three-sequential binding site model. Data fitting yielded estimated values for dissociation constants (*K*_D_^i^) and enthalpy variations (ΔH^i^) for each (i) binding site. Data in Tables 3 and 4 are presented as the average ± SD of 2 to 4 independent technical repetitions.

### ANS–fluorescence spectroscopy

Fluorescence spectra were collected at 37 °C with a Jasco FP 750 spectrofluorometer, with excitation and emission bandwidths set to 5 nm; each reported spectrum is an average of three accumulations.

The hydrophobic fluorescent probe ANS was diluted to 30 μM in 20-mM Tris HCl, pH 7.5, 150-mM KCl, 1-mM DTT buffer and incubated for 2 min with 2-μM GCAP2 variants. Emission fluorescence spectra were recorded in the 400 to 650-nm range after excitation at 380 nm in the presence of 500-μM EGTA and after sequential additions of 1-mM Mg^2+^ and 1-mM Ca^2+^.

### GC activity assays

Recombinant human guanylate cyclase 1 (GC1, alternatively GC-E, ROS-GC1, or RetGC1) was coexpressed with GFP in HEK293 cells as reported previously (74) to obtain a stable expression system. Briefly, fluorescent HEK293–GC cells were cultured in Dulbecco's modified Eagle's medium supplemented with fetal bovine serum (10%, v/v), penicillin (100 units/ml), streptomycin (100 μg/ml), and geneticin (500 μg/ml). HEK293 cells were transfected using branched polyethylenimine (10 kDa), and transfection was verified by Western blot analysis.

GC activity was measured after reconstitution of cell membranes as described previously (4, 33). Briefly, GC1 was incubated for 10 min at 30 °C with 5-μM GCAP1 (prepared as in Marino *et al*. (33)) or mGCAP2/nmGCAP2 or G157R variant and 1-mM free Mg^2+^, in the absence (<19 nM) or in the presence of Ca^2+^ (∼30 μM). Production of cGMP was analyzed by loading the reaction mixture onto a C18 reverse phase column (Lichrospher 100, RP-18, 5 mm, 250-4 (Merck)). Free Ca^2+^ concentration was adjusted using Ca^2+^–EGTA buffer solutions as described previously (26). Data presented in Table 5 represent the average ± SD two to three independent technical replicas.

### Homology model of human GCAP2

The three-dimensional model of human GCAP2 shown in Figure 1 (residues 1–186) was built using the software Prime (Schrödinger, LLC) using the NMR structure of bovine nmGCAP2 in its Ca^2+^-bound state as a template (PDB entry: 1JBA (8)) following the identification of globally conserved residues by the HMMER/Pfam algorithm. Loops were refined by the computational protocol described in Jacobson *et al*. (75). *In silico* mutagenesis to achieve the G157R substitution was performed using the BioLuminate suite, and both models (WT and G157R) were energy-minimized by performing four iterations, each consisting of 65 steps with root mean square gradient for convergence set to 0.01 kcal/mol/Å.

### Statistical data analysis

The statistical significance of the differences in hydrodynamic diameter and cGMP synthesis was assessed using a one-tailed *t* test (*p*-value = 0.001), where the null hypothesis was that the mean of a population was greater than or equal to the mean of the other population.

## Data availability

All data contained in the article are located in within the article.

## Supporting information

This article contains supporting information.

## Conflict of interest

The authors declare that they have no conflicts of interest with the contents of this article.
