# Supplementary material for: Molecular properties of human guanylate cyclase–activating protein 2 (GCAP2) and its retinal dystrophy–associated variant G157R
Source: J Biol Chem. 2021 Apr 1;296:100619. doi: 10.1016/j.jbc.2021.100619 (PMC8113879; doi:10.1016/j.jbc.2021.100619)
Supplement: Figures S1 to S3 and Tables S1 and S2 [file mmc1.pdf]

Supporting Information

Molecular properties of human guanylate cyclase-activating protein 2 (GCAP2) and its retinal dystrophy-associated variant G157R

by

Anna Avesani, Valerio Marino, Serena Zanzoni, Karl-Wilhelm Koch and Daniele Dell'Orco

This Supporting Information file contains the following items:

- Table S1
- Table S2
- Figure S1
- Figure S2
- Figure S3

Table S1. Estimation of the molecular mass (MM) of apo GCAP2 variants by Protein Calculator v3.4 (http://protcalc.sourceforge.net/) based on the canonical protein sequence (Uniprot entry: Q9UMX6) and comparison with MALDI-TOF mass spectrometry.

Variant	Theoretical MM (Da) (apo-forms)	MALDI-TOF MM (Da)
	**Monomer**	
**nmGCAP2**	23288.4	23375.1
**mGCAP2**	23499.8	23587.3
**G157R**	23598.9	23700.0
	**Dimer**	
**nmGCAP2**	46576.8	46840.9
**mGCAP2**	46999.6	47305.6
**G157R**	47197.8	47461.3

Table S2. Results from ANS fluorescence experiments. ΔI_{max} is calculated as $(I_{max}^{ion}-I_{max}^{EGTA})/I_{max}^{EGTA}$. $\Delta\lambda_{max}$ is calculated with respect to the protein in the apo form

Protein	State	I_{max} (A.U.)	$\Delta I_{max}/I_{max}$ (%)	λ_{max} (nm)
**nmGCAP2**	EGTA	329.3	-	488
	Mg^{2+}	330.7	0.4	488
	Mg^{2+} + Ca^{2+}	550.2	67.1	487
**mGCAP2**	EGTA	342.7	-	484
	Mg^{2+}	410.2	19.7	485
	Mg^{2+} + Ca^{2+}	645.8	88.4	485
**G157R**	EGTA	583.3	-	484
	Mg^{2+}	618.4	6.0	484
	Mg^{2+} + Ca^{2+}	751.2	28.8	485

Figure S1

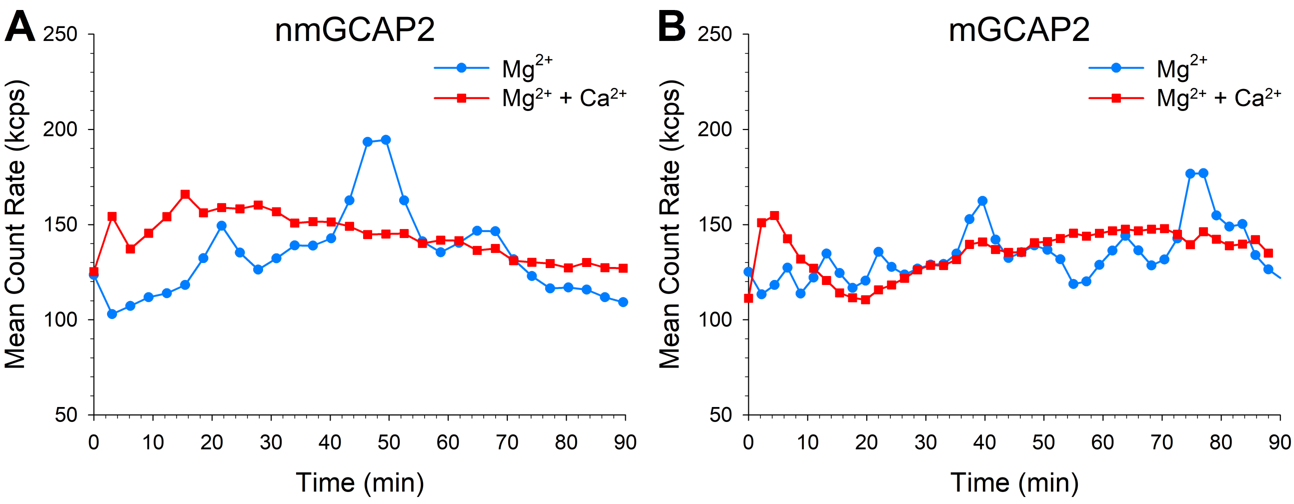

Figure S1. Time evolution of mean count rate assessed by dynamic light scattering. Mean Count Rate evolution over 90 min of ~40 μM protein sample (nmGCAP2 (A) and mGCAP2 (B)) in the presence of 500 μM EGTA (black), 500 μM EGTA + 1 mM Mg^{2+} (blue) or 1 mM Mg^{2+} + 500 μM Ca^{2+}(red).

Figure S2

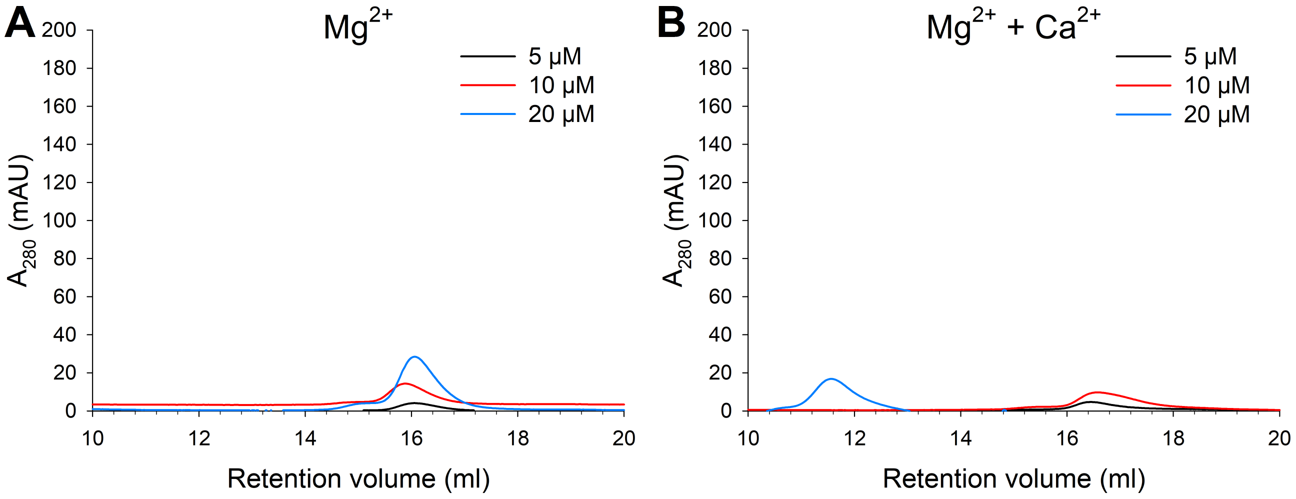

Figure S2. **Size-exclusion chromatography of the IRD-associated G157 variant.** SEC chromatograms of 5 µM (black), 10 µM (red) and 20 µM (blue) G157R in the presence of A) 1 mM Mg^{2+} and B) 1 mM Mg^{2+} + 500 µM Ca^{2+}.

Fig. S3

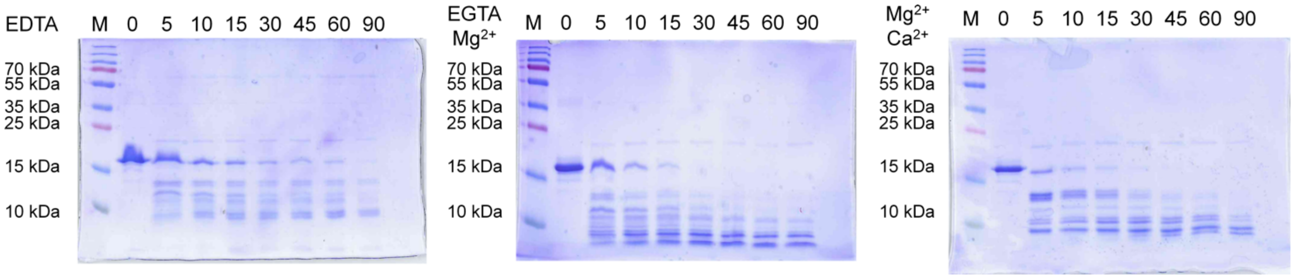

Figure S3. **Time-resolved proteolysis of nmGCAP2.** The protein was incubated with trypsin (60:1 molar ratio) in the presence of 1 mM EDTA (left), 500 µM EGTA + 1 mM Mg^{2+} (center) or 1 mM Mg^{2+} + 1 mM Ca^{2+}(right).
